# Failures in Business

**Published:** 1873-04

**Authors:** 


### FAILURES IN BUSINESS.

The man who has never failed in business cannot possibly know whether he is honest or not, cannot possibly know whether he has any "grit" in him or is worth a button. It is the man who fails and then rises who is really great in his way.

Peter Cooper failed in making hats, failed as a cabinet-maker, locomotive builder, and grocer; but as often as he failed he "tried and tried again," until he could stand upon his feet alone, then crowned his victory by giving a million dollars to help the poor boys in times to come.

Horace Greeley tried three or four lines of business before he founded the *Tribune*, worth to-day a million of dollars.

Patrick Henry failed at everything he undertook, until he made himself the orator of his age and nation.

The founder of the *Herald* kept on failing and sinking money for ten years, and then made the most valuable newspaper on earth.

Stephen A. Douglass made dinner tables and bedsteads and bureaus for many a long year before he made himself a "giant" on the floor of Congress.

Abraham Lincoln failed to make both ends meet by chopping wood, failed to earn his salt in the galley-slave life of a Mississippi flat-boatman; he had not even wit enough to run a grocery, and yet he made himself the grandest character of the nineteenth century.

General Grant failed at every thing, except smoking cigars; he learned to tan hides, but could not sell leather enough to purchase a pair of breeches; a dozen years ago he "brought up" on top of a wood pile, "teaming" it to town for forty dollars a month; and yet he is the greatest soldier of the age, and is now the honored head of a great nation, the choice of millions of voters. The lesson for every young man is this: As long as you have health and power to do, go ahead; if you fail at one thing, try another; and a third—a dozenth even. Look at the spider; nineteen times it tried to throw out its web to a place of attachment, and on the twentieth it succeeded. The young man who has the "gift of continuance" is the one whose foot will some day stand on high ground and will be able to breast the angry waters of human discouragement.

